# Image analysis as an adjunct to manual HER-2 immunohistochemical review: a diagnostic tool to standardize interpretation

**DOI:** 10.1111/j.1365-2559.2010.03577.x

**Published:** 2010-07

**Authors:** Lynne Dobson, Catherine Conway, Alan Hanley, Alex Johnson, Sean Costello, Anthony O’Grady, Yvonne Connolly, Hilary Magee, Daniel O’Shea, Michael Jeffers, Elaine Kay

**Affiliations:** SlidePath LtdDublin, Ireland; 1 Department of Histopathology, Beaumont Hospital and Royal College of SurgeonsDublin, Ireland; 2 Department of Histopathology, Adelaide and Meath incorporating National Children’s HospitalDublin, Ireland

**Keywords:** fluorescence *in situ* hybridization, HER-2, image analysis, immunohistochemistry, virtual slides

## Abstract

Dobson L, Conway C, Hanley A, Johnson A, Costello S, O’Grady A, Connolly Y, Magee H, O’Shea D, Jeffers M & Kay E (2010) *Histopathology***57,** 27–38 **Image analysis as an adjunct to manual HER-2 immunohistochemical review: a diagnostic tool to standardize interpretation**

**Aims:**

Accurate determination of HER-2 status is critical to identify patients for whom trastuzumab treatment will be of benefit. Although the recommended primary method of evaluation is immunohistochemistry, numerous reports of variability in interpretation have raised uncertainty about the reliability of results. Recent guidelines have suggested that image analysis could be an effective tool for achieving consistent interpretation, and this study aimed to assess whether this technology has potential as a diagnostic support tool.

**Methods and results:**

Across a cohort of 275 cases, image analysis could accurately classify HER-2 status, with 91% agreement between computer-aided classification and the pathology review. Assessment of the continuity of membranous immunoreactivity in addition to intensity of reactivity was critical to distinguish between negative and equivocal cases and enabled image analysis to report a lower referral rate of cases for confirmatory fluorescence *in situ* hybridization (FISH) testing. An excellent concordance rate of 95% was observed between FISH and the automated review across 136 informative cases.

**Conclusions:**

This study has validated that image analysis can robustly and accurately evaluate HER-2 status in immunohistochemically stained tissue. Based on these findings, image analysis has great potential as a diagnostic support tool for pathologists and biomedical scientists, and may significantly improve the standardization of HER-2 testing by providing a quantitative reference method for interpretation.

## Introduction

Targeted therapeutics or personalized medicine regimes are driving a new era of integrated diagnostics and therapeutics, particularly in oncology. Many anticancer antibodies have been approved in association with companion tests for biomarker expression to identify the most responsive patients, ensuring that accurate evaluation of biomarker status has become particularly acute in the clinical laboratory. The role that biomarkers can play is exemplified by HER-2: a prognostic, predictive and therapy selection factor for patients with breast cancer. Amplification of the *HER-2* gene or overexpression of its protein product in cell membranes is seen in 10–30% of invasive breast cancer and is associated with increased disease recurrence and poor prognosis.^1^–^5^ Clinically, HER-2 is important as the target of the monoclonal antibody trastuzumab (Herceptin®, Genentech, CA, USA), which significantly improves response rate, disease progression and overall survival when used in an adjuvant setting compared with chemotherapy alone.^6^–^8^ The association between HER-2 expression and Herceptin® response has led to the recommendation that this parameter should be evaluated in every primary invasive breast cancer to distinguish those patients for whom the drug may be of benefit, not only because of the expense of treatment, but also because of its potential to cause myocardial toxicity if incorrectly prescribed.^9^–^11^fc

The therapeutic relevance of HER-2 status demands highly reliable and robust testing to identify tumours that overexpress this protein.^12^ The recommended evaluation method for HER-2 is immunohistochemistry (IHC) to detect expression of the HER-2 protein in cell membranes, with equivocal cases confirmed at the gene expression level using fluorescence *in situ* hybridization (FISH).^9^,^11^ FISH is considered the gold-standard method of evaluation, affording an objective and quantitative scoring system; however, this technique suffers from fading fluorochromes and thus poor long-term stability, in addition to a requirement for specialized microscopic equipment that restricts its use in conventional laboratories.^8^,^12^ Chromogenic *in situ* hybridization (CISH) or silver-enhanced *in situ* hybridization, which do not rely on fluorescent microscopy, represent alternatives to FISH in terms of *HER-2* oncogene analysis. However, although these methods have been determined to give comparable results to FISH, they are not yet widely utilized in diagnostic pathology.^11^,^13^

In contrast, qualitative IHC testing is advocated as the primary assay for identifying candidates for trastuzumab because it is readily available, easily performed in most clinical pathology laboratories and has many advantages over FISH or CISH in terms of economics, as well as being highly amenable to automation.^8^,^10^,^14^,^15^ Nonetheless, despite efforts to standardize assay protocol and interpretation, antibodies and methods vary across laboratories and IHC scoring remains an inherently subjective process to which only limited statistical confidence can be assigned due to inherent observer variability and the semiquantitative nature of the data.^8^,^10^,^16^–^19^ Even for the trained eye of a pathologist, accurate distinction between the nominal categories (0, 1+, 2+, 3+) is difficult and often arbitrary, and significant variation is introduced as a result of overusing the intermediate category during reviews.^2^,^20^

Recently, high rates of discordance between IHC reviewed at high-volume HER-2 reference centres and low-volume regional laboratories has cast doubt on the reliability of results.^5^,^21^–^23^ As this stands alone in determining which patients are likely to respond to trastuzumab therapy, additional attention to the performance and interpretation of IHC testing is now warranted.^24^,^25^ Participation in external quality assurance (EQA) schemes is recommended and, according to the updated National Comprehensive Cancer Network guidelines, if standards cannot be met material should be sent to a reference laboratory. Nonetheless, although the current EQA schemes assess methodologies, they do not attend to disparity in interpretation; in order to address this the American Society of Clinical Oncology has suggested that image analysis could be an effective tool for achieving consistency.^9^ Indeed, virtual pathology, the process of assessing digital images of histology slides, is gaining momentum in today’s laboratory environment, with digital image acquisition systems commonplace and associated image analysis solutions viewed by most as the next critical step.^26^ Image analysis may serve to reduce scoring variability by providing a quantitative HER-2 reference tool, thus standardizing the evaluation system.

Thus there is an urgent need to develop more sensitive image analysis tools that may be utilized with any of the prevalent HER-2 antibody kits in clinical pathology. Towards this goal, we have developed a HER-2 image analysis algorithm that may be applied to slides immunohistochemically stained with Dako HercepTest® (Dako, Glostrup, Denmark), Ventana Pathway® (Ventana, Tucson, AZ, USA) or Leica Oracle™ (Leica, Heerbrugg, Switzerland) HER-2 antibodies. In particular our approach employs a novel quantification base, determining the continuity or extent of circumferential membranous immunoreactivity, a parameter overlooked by other algorithms despite being an important factor for differentiating HER-2 classification. It is hypothesized that by considering this parameter, the number of ambiguous cases may be reduced by enabling better separation between negative and equivocal groups. This multi-site study set out to assess whether this HER-2 algorithm could improve the diagnostic accuracy of IHC scoring, with the performance of the algorithm measured against both manual review and FISH evaluation as the accepted standard.

## Materials and methods

### Slide preparations

A total of 448 consecutive cases were selected for this study from the archives of Beaumont Hospital and Adelaide and Meath Hospital, two HER-2 reference laboratories in Dublin, Ireland, of which 425 were successfully stained, reviewed and digitized. All cases were formalin fixed, paraffin embedded and processed in the routine diagnostic laboratory of the institute of origin according to standardized protocols.

#### Immunohistochemistry

The cases supplied from Beaumont Hospital were assessed for HER-2 protein expression using Dako HercepTest® (*n* = 144) and Leica Oracle™ HER-2 (*n* = 140) antibodies according to the manufacturer’s instructions. Those cases supplied from Adelaide and Meath Hospital were analysed for HER-2 protein expression with Ventana Pathway® HER-2 (4b5) (*n* = 141) according to the manufacturer’s instructions. In all cases, suitable negative and positive control slides were treated in a similar manner to ensure appropriate staining.

#### Fluorescence in situ hybridization

A representative cohort of cases was selected for FISH testing for verification purposes. Of the 425 cases supplied, 219 were analysed for *HER-2* gene amplification using the PathVysion® HER-2 DNA probe kit and paraffin wax pretreatment kit (Vysis Inc., Queenborough, UK) in the facility of origin. All procedures were performed in accordance with the manufacturer’s recommended protocol.

#### Digitization of slides and archival of images

Immunohistochemically stained full-face sections were digitized by SlidePath using a NanoZoomer Digital Pathology (NDP) System (Hamamatsu, Welwyn Garden City, UK). The NDP system utilizes charge-coupled device time delay integration technology to achieve scans with a spatial resolution of 0.46 μm/pixel. Scanning time at 20× was approximately 3 min for a 20 × 20 mm biopsy specimen. Images were approximately 55–487 Mb per whole section biopsy specimen and were archived using SlidePath’s Digital Slideserver, a secure, web-enabled digital slide management system.

### Manual evaluation of HER-2 status

At each site, HER-2 protein expression was reviewed by a Consultant Pathologist. For those cases where FISH analysis was carried out, gene amplification status was reviewed by a Biomedical Scientist. All cases were classified according to the new American Society of Clinical Oncology/College of American Pathologists (ASCO/CAP) and UK guideline recommendations for HER-2 testing as detailed in Table 1.

**Table 1:** American Society of Clinical Oncology/College of American Pathologists and UK guideline recommendations for HER-2 classification^9^,^11^

Classification	HER-2 grade	IHC staining pattern	FISH criteria
Negative	0/1+	No staining or weak, incomplete membranous staining in <10% of tumour cells	HER2/Chr17 ratio <1.8
Equivocal	2+	Weak to moderate complete membranous staining that is non-uniform or weak in intensity in at least 10% of cells	HER2/Chr17 ratio between 1.8 and 2.2
Positive	3+	Uniform, intense membranous staining in >30% of tumour cells	HER2/Chr17 ratio >2.2

### Image analysis classification of HER-2 status

#### Tissue IA system

**Figure 1:**
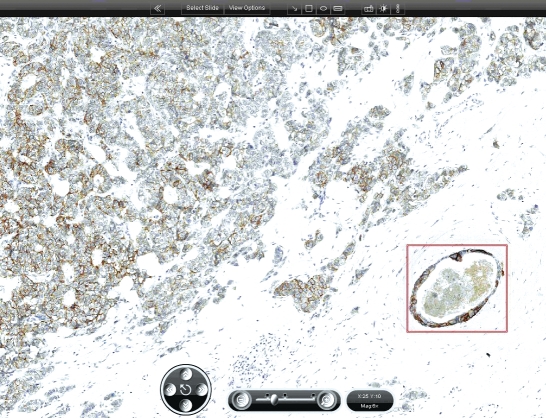
As a prerequisite for image analysis and in accordance with clinical guidelines, the non-invasive regions of tumour were annotated online by a Pathologist and excluded from analysis.

The HER-2 image analysis algorithm was deployed within SlidePath’s Tissue IA system, a web-enabled image analysis solution for the interpretation of virtual slides. As a prerequisite for image analysis, invasive tumour regions of all cases were annotated on-line by the respective Consultant Pathologist, with non-invasive or ductal carcinoma *in situ* regions excluded from analysis (Figure 1).

Entire full-face sections or annotated regions of these cases were subsequently submitted for batch image analysis. Tissue IA employs a grid computing model that distributes image data across multiple processing nodes, facilitating high-throughput automated analysis of virtual slides. The HER-2 algorithm utilizes a specific colour definition file to define immunopositive tissue within an image and isolates the cell membrane using edge detection techniques. The output from the algorithm includes a number of quantitative measurements such as membrane staining absorbance, percent membrane positive pixels in tissue and percent membrane continuity.

#### Generation of probability classifier

From the total cohort of 425 cases, a training set of 150 cases containing an equal distribution of slides stained with Ventana Pathway®, Leica Oracle™ and Dako HercepTest® antibodies was randomly chosen by assigning cases with a random real number ≥0 and <1 and selecting the 50 highest numbers for each antibody cohort. Table 2 illustrates the distribution of cases according to the manual review in the training and validation sets.

**Table 2:** Distribution of cases in training and validation sets

	Training set		Validation set	
	Number of cases	% of total	Number of cases	% of total
0/1+	89	59.4	183	66.5
2+	32	21.3	40	14.5
3+	29	19.3	52	19.0
Total	150	100	275	100

The image analysis results for these slides were exported for statistical analysis and were used to generate a probability classifier, which determined a dedicated HER-2 score (0/1+, 2+ or 3+) based on the distribution of staining absorbance and membrane continuity for each category. In addition, a constraint was included that automatically defined any case with <1% immunopositive pixels in selected regions as negative or 0/1+. These computational steps were then incorporated into the algorithm, resulting in an output of a dedicated HER-2 classification, along with a percentage confidence in that score.

#### Validation of cell-line standards

For 180 of the 275 remaining test cases the manufacturer control cell line material was also available for analysis. Cell lines provide consistency in terms of both the quantity of material and the gradation of protein expression, and when used as part of a validated system have applications in internal quality assurance, providing a standard against which a laboratory can gauge against day-to-day drift in assay sensitivity.

### Statistical analysis

Sigma Plot Version 8 (SPSS Inc., Chicago, IL, USA) and SPSS Version 15 (SPSS Inc.) were used to perform statistical analyses including concordance and Cohen’s κ statistics. The Landis and Koch Kappa interpretation scale was used to evaluate the level of κ agreement.^27^

The sensitivity and specificity of both the automated and manual review were calculated using FISH evaluation as the gold standard, where:


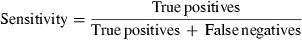
(1)

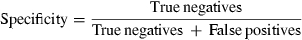
(2)

## Results

### Concordance with manual review

**Figure 2:**
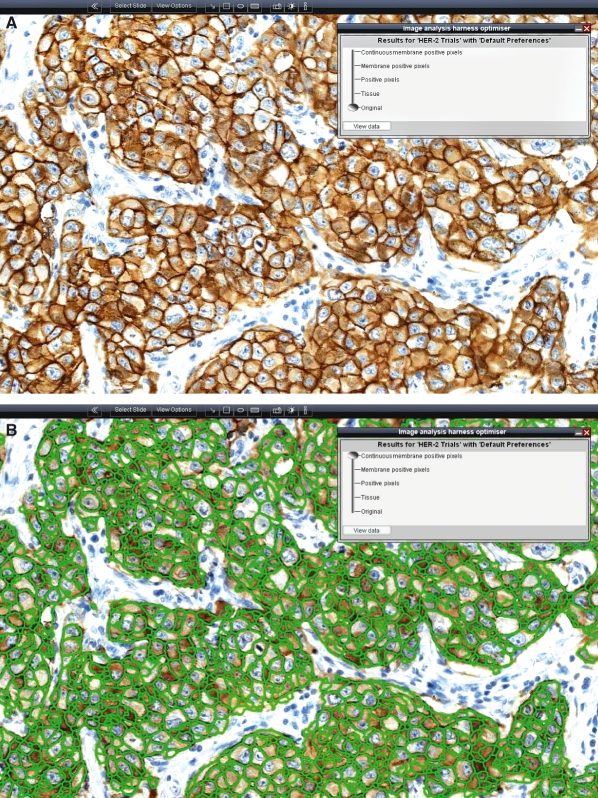
**A**, Unprocessed image of breast tissue that has been immunohistochemically stained with antibodies probing for HER-2 protein expression. **B**, Areas detected as positive for continuous membranous immunoreactivity by image analysis are highlighted in green.

The concordance between image analysis evaluation of HER-2 status and manual review by a Consultant Pathologist was blindly assessed on a cohort of 275 cases stained with Dako HercepTest®, Leica Oracle™ and Ventana Pathway®. Statistical analysis established that there was agreement in the classification of 250 of the 275 cases, representing a concordance of 91% between the pathology and image analysis reviews (Table 3). Kappa was evaluated to be 0.81, which indicates ‘almost perfect’ agreement between manual review by a pathologist in a reference laboratory and automated review using image analysis. Table 3 also reveals that in this study image analysis reported a lower number of equivocal cases than the manual pathology review. Indeed, of the 17 cases reclassified by image analysis, 15 had been FISH tested and in each of these cases the gene amplification status was concordant with the reclassified score by image analysis, suggesting that image analysis would have led to a significant cost saving in this instance. Figure 2 shows representative images from the system and illustrates the ability of the HER-2 algorithm to detect regions of positively and continuously immunoreactive cell membrane.

**Table 3:** Performance of image analysis with clinical samples assessed on the basis of the American Society of Clinical Oncology/College of American Pathologists and UK scoring guidelines

		Image analysis classification			
		0/1+	2+	3+	Total
Manual classification	0/1+	178	5	0	183
	2+	15	23	2	40
	3+	0	3	49	52
Total		193	31	51	275

Concordance, 90.9%.

κ, 0.811.

As expected, analysis of the corresponding cell-line control material determined that those slides stained using automated systems exhibited less variance than those prepared manually. Nonetheless, normalization to compensate for variance had no impact on the classification of HER-2 by image analysis.

### Concordance with FISH evaluation

A number of cases in the study (*n* = 136) were also analysed by FISH, the ‘gold-standard’ method of HER-2 evaluation. The concordance rate between *HER-2* gene amplification and IHC review was determined to be excellent for both image analysis and the pathology review, demonstrating that image analysis can robustly and accurately classify HER-2 status (Table 4). However, it was observed that image analysis review of the IHC sections attained a slightly higher concordance rate with FISH than the manual review (95% versus 92%, respectively). Although both methods correctly classified 13 FISH+ cases as 3+ IHC cases, quantification by image analysis identified 92 cases with no gene amplification, in comparison with 83 for the pathology review. This was attributed to improved differentiation between negative and equivocal cases by image analysis and suggests that the automated method of review is more accurate than visual scoring.

**Table 4:** Concordance between *HER-2* gene amplification and HER-2 protein expression reviewed by a pathologist and by image analysis

	IHC					
	Manual classification			Image analysis classification		
FISH	Negative (0/1+)	Positive (3+)	Equivocal (2+)	Negative (0/1+)	Positive (3+)	Equivocal (2+)
Positive	7	13	7	6	13	8
Negative	83	1	25	92	0	17
Concordance	92%	93%	NA	94%	100%	NA

**Figure 3:**
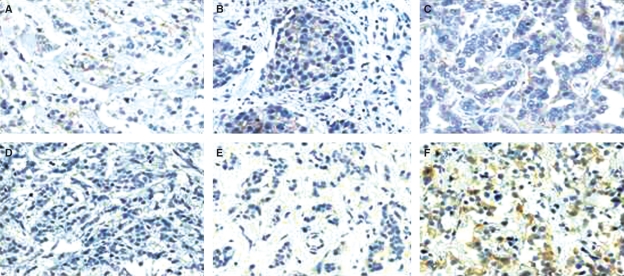
Cases where IA and Manual review agreed on a classification of 0/1+ but gene amplification was determined to be positive by fluorescence *in situ* hybridization. Cases were stained using HER-2 antibodies from: **A**, Ventana Pathway®; **B**, Ventana Pathway®; **C**, Ventana Pathway®; **D**, Dako HercepTest®; **E**, Leica Oracle™; **F**, Leica Oracle™.

Nonetheless, it is evident from Table 4 that significant disagreement between IHC classification and FISH amplification occurred in six cases, which were subsequently re-examined for possible causes of conflicting results. Figure 3 illustrates that IHC staining was negligible in all of these cases and the pathology and image analysis reviews agreed that these should be categorized as negative or 0/1+. However, in each case gene amplification was determined to be positive by FISH, suggesting these are false-negative IHC cases. A number of previous studies have reported this phenomenon in approximately 7% of HER-2 FISH+ results, which would correlate with the figures determined here, and the cause is generally attributed to destruction of the HER-2 epitope or antigen loss during fixation or processing.^3^,^28^ It was noted that if these slides were omitted from Table 4 the sensitivity of both review methods would be significantly improved, with HER-2 classification by image analysis review achieving 100% sensitivity and specificity.

**Figure 4:**
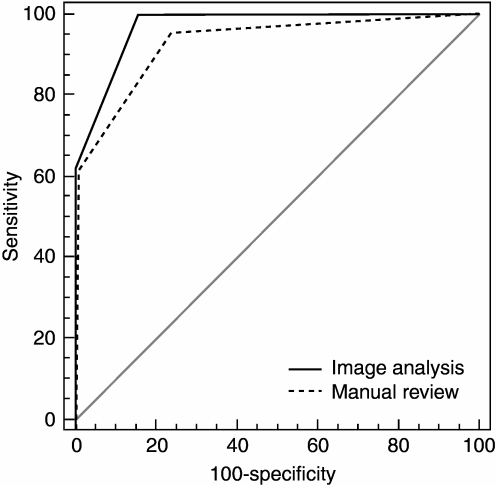
Receiver–operator curve for the manual and image analysis review of 136 informative cases (a curve reaching the upper left corner implies better performance).

Receiver–operating characteristic curve analysis was used to compare the accuracy of the manual and image analysis methods with FISH evaluation as the standard (Figure 4). The area under the curve value was found to be 0.93 [95% confidence interval (CI) 0.867, 0.965] for the manual review with 0.97 (95% CI 0.925, 0.992) obtained by image analysis, confirming the accuracy of the automated algorithm. Both review methods were found to be statistically significant (*P* < 0.0001).

### Comparison with other commercially available image analysis systems

A number of other image analysis systems are commercially available for use as decision support tools in the clinical setting. Nevertheless, Table 5 illustrates that the accuracy in predicting HER-2 status varies considerably across the offerings and a number of key distinguishing factors exist between the systems. In comparison with the data submitted by other systems for Food and Drug Administration (FDA) approval, this validation study across a larger cohort of clinical cases has established that Tissue IA achieved a 5–14% higher correlation with manual review. Indeed, the 91% concordance rate reported here is substantially greater than the 70% agreement detailed by Camp *et al.*,^2^ 2003, using the automated quantitative analysis (AQUA) system. In addition, the performance of the HER-2 algorithm under trial has attained high levels of concordance with gene amplification status, greater than that reported for the automated cellular imaging system (ACIS) by Tawfik *et al.* and Wang *et al.*^29^,^30^ Furthermore, this system has been validated to perform with slides stained using Dako HercepTest®, Leica Oracle™ and Ventana Pathway® HER-2 antibodies.

**Table 5:** Comparison of SlidePath’s Tissue IA system with other commercially available systems for HER-2 analysis

Manufacturer	SlidePath	Aperio	BioImagene	Dako (Chromavision)	Genetix (Applied Imaging)	Ventana (TriPath Imaging)	
System	Tissue IA	Scanscope XT	Pathiam	ACIS	Ariol	VIAS	
Assay	Dako HercepTest® Leica Oracle Bond™ Ventana Pathway® (4b5)	Dako HercepTest®	Dako HercepTest®	Dako HercepTest®	Dako HercepTest®	Ventana Pathway® (4b5)	Ventana Pathway® (cb11)
Concordance with manual review (sample size)	91% (*n* = 275)	86%* (*n* = 180)	81%* (*n* = 176)	75%* (*n* = 90)	a* (*n* = 124)	86%* (*n* = 206)	77%* (*n* = 201)
Image format support	•	○	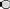	○	○	○	
Dependence on manual selection	•	•	•	•	•	•	
Quantitation base	Intensity, continuity	Intensity	Morphology, intensity	Intensity	Intensity	Intensity	

High •, Intermediate 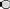, Low ○.

* Data from Food and Drug Administration 510 k substantial equivalence reports (http://www.fda.gov).

a: The likelihood of the image analysis system producing a consistent score on a given slide is as likely as the pathologists are to agree with each other.

**Figure 5:**
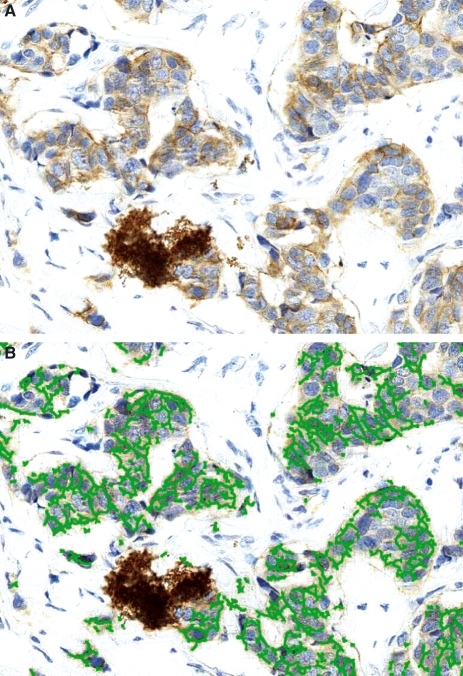
**A**, Artefactual chromogenic staining and **B**, the ability of the HER-2 algorithm to identify and exclude these areas from analysis.

The disparity in the accuracy of the image analysis systems may be attributed to a variety of factors. In the first instance, the HER-2 algorithm reviewed here has the capacity to identify and eliminate artefactual staining which may be observed when automated staining systems are employed or improper use of such equipment occurs (Figure 5). Without this functionality, true-negative cases may be incorrectly classified by image analysis as equivocal due to the high intensity of immunoreactivity.

**Figure 6:**
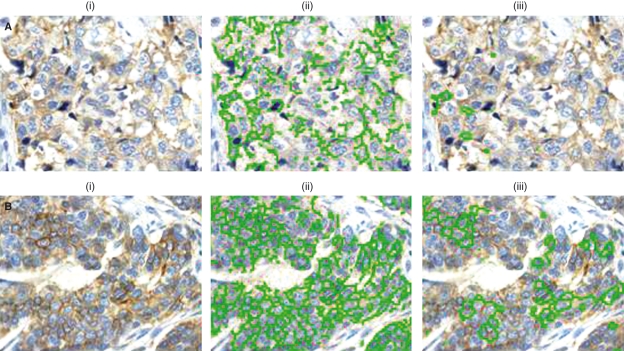
Importance of evaluating circumferential membranous immunoreactivity, which enables differentiation of 1+ and equivocal (2+) cases by image analysis. **A**, 1+ in-house control tissue [fluorescence *in situ* hybridization (FISH) score 1.18]. **B**, 2+ in-house control tissue (FISH score 1.97); (i), original image; (ii), regions of issue identified as immunopositive membrane; (iii), regions of positively and continuously immunoreactive membrane.

Moreover, it is evident from Table 5 that the distinguishing factor between the image analysis systems is the quantification base used to determine the extent of HER-2 protein expression. While all algorithms quantify the intensity of membranous immunoreactivity, the algorithm under trial also determines the continuity of membranous reactivity, the parameter that underpins the definition of positive HER-2 status. Although intensity of reactivity is critical for distinguishing the 3+ cases, consideration of membrane continuity is essential for clear distinction of the 0/1+ and the equivocal 2+ categories. Indeed, Figure 6 demonstrates that although the intensity of membranous immunoreactivity can appear to be similar for both groups, the extent of continuity of that reactivity is undoubtedly a distinguishing factor that enables correct differentiation of a number of ambiguous visual IHC scores.

## Discussion

High levels of HER-2 protein expression or *HER-2* gene amplification are used to identify patients for whom trastuzumab may be of benefit for treatment of breast cancer in the metastatic or adjuvant disease settings.^28^ In accordance with the HER-2 testing guidelines, in most laboratories IHC is carried out first with additional testing accomplished by FISH. However, assignment of HER-2 grade by assessment of IHC is inherently subjective and dependent on the skill and experience of the reviewing pathologist.^31^ Thus the standardization of diagnosing breast cancer is a very important task for improving personalized cancer patient care, as a cancer patient to whom an inappropriate drug is given will face disease progression during the treatment time impacting on overall survival rate and increased costs.^32^

The last 10 years have seen enormous advances in the capabilities of image analysis systems applied to tissue sections with complex computer algorithms used to interpret the images.^26^ Digital microscopy is increasingly being used to document and analyse tissue specimens in modern research laboratories and it has recently been proposed that newly introduced image analysis technology has a major role to play in the progress of diagnostic pathology.^12^,^33^ In comparison with human-based assessment, automated image analysis offers numerous advantages such as precise, reproducible, continuous and objective assessment of protein expression.^17^,^34^ Indeed, image analysis has been used to evaluate the expression of nuclear markers such as oestrogen and progesterone receptor; cytoplasmic markers such as β-catenin; and other membrane proteins such as E-cadherin.^35^,^36^–^38^ Nonetheless, a major requisite for the acceptance of image analysis in the clinical laboratory is that it must yield high concordance with the current gold standard method. Indeed, although the ASCO/CAP guidelines have advocated the use of image analysis for HER-2, a degree of resistance to its adoption in the clinical setting has been observed, perhaps due to the low accuracy and restrictions of the currently available and approved systems.

The approach detailed in this study has aimed to address the inherent deficiencies in other systems. In the first instance, the algorithm under review measures the continuity of membranous immunoreactivity as well as the intensity of reactivity, and has demonstrated that consideration of both parameters enables accurate distinction of HER-2 status. Furthermore, this HER-2 algorithm has been validated to perform with some of the most prevalent HER-2 antibodies on the market. Although the HER-2 guidelines for testing do not stipulate the use of a particular antibody, the Dako HercepTest® and Ventana Pathway® are recommended as FDA-approved kits, and the Leica Oracle™ HER-2 antibody is also frequently employed in laboratories that have demonstrated concordance with a validated method.

Our findings demonstrate that image analysis can accurately and robustly classify HER-2 status. A concordance rate of 91% was observed in comparison with manual review by a pathologist, and the significant value of image analysis was exemplified by a 4% reduction in the reporting of equivocal cases. This represents a decrease in the number of cases requiring confirmatory FISH testing and thus a potential cost saving for clinical laboratories. Moreover, the concordance of image analysis with gene amplification status as the standard was observed to be 95%, which represents better correlation and accuracy with FISH than the manual interpretation of IHC. The data from this study are substantially greater than reported by existing systems in FDA approval documentation and independently by Camp *et al.* using the AQUA platform, or Wang *et al.* and Tawfik *et al.* using the ACIS system.^2^,^29^,^30^ Indeed, in comparison with FISH, the ACIS system was demonstrated to falsely predict 4–11% of cases as HER-2 amplified, which could have a significant impact on patient welfare.^29^,^30^ In contrast, the platform reviewed here accurately predicted all *HER-2* gene-amplified cases with a false-positive rate of 0%.

Although it is generally accepted that the standard assessment of IHC will remain the manual pathology review, our findings suggest that integration of image analysis into the diagnostic workflow could significantly enhance the reproducibility of scoring, particularly in those laboratories where there is lack of experience in interpreting HER-2 staining. However, aside from providing assistance for interpretation, image analysis could be utilized as an internal resource to qualify the quality of IHC, introducing an unprecedented level of internal laboratory quality assurance. Both the ASCO/CAP and UK guidelines recommend the use of control material, which should be used consistently by each laboratory with each run of tests.^9^,^11^ Although tissue controls are often employed, this material is frequently difficult to acquire and can exhibit variation in tumour expression and fixation that is far from ideal.^39^ In contrast, cell lines with differing but constant levels of HER-2 expression have been advocated as standard material against which assay sensitivity can be gauged.^39^ Image analysis of cell line standards could be utilized to assess batch variability of staining within clinical laboratories, providing a means to validate the ability of a laboratory to produce consistently immunostained slides, flagging batches should intensity of reactivity fall outside of acceptable limits. This may be of interest to many external quality assurance providers such as CAP, UK National External Quality Assessment Service (NEQAS), Nordic Immunohistochemical Quality Control and Royal College of Pathologists of Australasia (RCPA) Quality Assurance Program (QAP), as it will enable laboratories to grade their performance against other facilities using the same standards and antibodies, similar to the annual performance rankings generated by the RCPA Anatomical Pathology QAP. Indeed, whilst organizations such as UK NEQAS have a role to play in ensuring a high standard of quality assessment, at present UK NEQAS schemes focus primarily on methodologies rather than the interpretation of results.^40^ Nonetheless, Walker *et al.*^11^ documented that ‘virtual systems are being explored’ and it is likely that image analysis will play a significant role in the provision of quality assurance schemes in the future.

Undoubtedly, the recent reports of poor observer variability regarding the evaluation of HER-2 in the clinical setting justify the development of software tools to help standardize interpretation, particularly in equivocal cases. Based on this study, Tissue IA has been validated as a consistent scoring tool with excellent levels of concordance with manual scoring and FISH, advocating the use of Tissue IA as a decision support system for pathologists to assist in the diagnosis of disease. Further independent studies to demonstrate the accuracy of the system have been initiated.

## Intended use

SlidePath applications are not cleared by the FDA, Health Canada, or in the EU for diagnostic or clinical use. All applications are intended solely for use in the research or educational setting, such as university or pharmaceutical development. These applications are described as Research Applications or Research Use Only.

## Abbreviations:

ACIS: automated cellular imaging system
AQUA: automated quantitative analysis
ASCO: American Society of Clinical Oncology
CAP: College of American Pathologists
CI: confidence interval
CISH: chromogenic *in situ* hybridization
EQA: external quality assurance
FDA: Food and Drug Administration
FISH: fluorescence *in situ* hybridization
IA: image analysis
IHC: immunohistochemistry
NDP: NanoZoomer Digital Pathology
NEQAS: National External Quality Assessment Service
QAP: Quality Assurance Program
RCPA: Royal College of Pathologists of Australasia
